# Yield, Essential Oil and Quality Performances of *Artemisia dracunculus*, *Hyssopus officinalis* and *Lavandula angustifolia* as Affected by Arbuscular Mycorrhizal Fungi under Organic Management

**DOI:** 10.3390/plants9030375

**Published:** 2020-03-18

**Authors:** Nadezhda Golubkina, Lidia Logvinenko, Maxim Novitsky, Svetlana Zamana, Sergey Sokolov, Anna Molchanova, Oksana Shevchuk, Agneszka Sekara, Alessio Tallarita, Gianluca Caruso

**Affiliations:** 1 Agrochemical Research Center, Federal Scientific Center of Vegetable Production, 143072 Moscow, Russia; vovka_ks@rambler.ru; 2 Nikita Botanic Gardens, National Scientific Center of the RAS, 298648 Yalta, Russia; logvinenko-1963@list.ru (L.L.); maxim.novickiy@bk.ru (M.N.); oksana_shevchuk1970@mail.ru (O.S.); 3 Department of Agriculture and Crop Production, State University of Land Management, Kazakova str. 15, 10506 Moscow, Russia; svetlana.zamana@gmail.com; 4 Scientific Technical Center ‘Sustainable Development of Agroecosystems’, 143072 Moscow, Russia; sergey.alex.sokolov@gmail.com; 5 Department of Vegetable and Medicinal Plants, University of Agriculture, 31-120 Krakow, Poland; agnieszka.sekara@urk.edu.pl; 6 Department of Agricultural Sciences, University of Naples Federico II, 80055 Portici, Naples, Italy; lexvincentall@gmail.com (A.T.); gcaruso@unina.it (G.C.)

**Keywords:** AMF, aromatic plants, plant biomass, oil components, mineral nutrients, stress tolerance, antioxidants

## Abstract

Utilization of arbuscular mycorrhizal fungi (AMF) for enhancing growth and development as well as production of essential oil in aromatic plants has been increasingly drawing research interest. In order to assess the AMF effects on different aromatic species, an open-field experiment was carried out using *Artemisia dracunculus* (tarragon), *Lavandula angustifolia* (lavender) and *Hyssopus officinalis* (hyssop). AMF stimulated the growth of tarragon and lavender plants, whereas hyssop showed a slight developmental slowing; nonetheless, a significant increase in essential oil content in the three species was seen. AMF application increased the biomass of *A. dracunculus* and *H. officinalis* by 20–35%. No differences in antioxidant activity and phenolics content were recorded at harvest between the control and AMF-inoculated plants, but the latter showed a significant increase in antioxidant status upon storage at high temperature and humidity compared to the untreated control. The enhancement of abiotic stress resistance during storage in plants inoculated with AMF was the highest in *A. dracunculus*, and the lowest in *H. officinalis*, while the untreated control plants showed a significant decrease in phenolics, ascorbic acid and chlorophyll content, as well as antioxidant activity, upon the abiotic stress. AMF inoculation differentially affected the mineral composition, increasing the accumulation of Se, I and Zn in *A. dracunculus*, and decreasing the levels of heavy metals and Co, Fe, Li, Mn in *H. officinalis*. Based on the outcome of the present research, AMF inoculation resulted in a significant enhancement of the overall performances of *A. dracunculus*, *L. angustifolia* and *H. officinalis*, and also in the improvement of plant antioxidant status upon storage in stress conditions.

## 1. Introduction

Among the modern environmentally friendly technologies for plant production, the utilization of arbuscular mycorrhizal fungi (AMF) is one of the most promising, especially in organic management [1]. Indeed, the latter approach results in the improvement of plant and water uptake as a result of higher root system expansion, optimization of protection against biotic and abiotic stresses and enhancement of plant antioxidant status [2,3]. Interestingly, AMF establish symbiotic associations with more than 80% of terrestrial plants, and the rate of their colonization is reportedly dependent on either fungi and plant species or environmental factors (e.g., soil characteristics) [4]. The increased accumulation of macro-elements (mainly N and P) and micro-elements (Zn, S, Cu, Fe, and Mn) upon AMF inoculation are highly important in producing vegetable and fruit crops as well as medicinal herbs [5].

Several studies demonstrated the possibility of the significant enhancement of chlorophyll, anthocyanins, polyphenols and, in particular, essential oil content in aromatic plants [3]. Increased nutrient concentration, plant biomass and essential oil content, as well as glomalin-related soil proteins were recorded in AMF-inoculated *Pelargonium graveolens* [6]. AMF application promoted essential oil synthesis in thyme, sage, oregano [7], basil [8,9], *Artemisia annua* [10] and menthol mint [11].

Despite the recognized important role of AMF in plant nutrition and growth under organic management, the effect of arbuscular mycorrhizal fungi on the accumulation of secondary metabolites and mineral composition in plants has not been widely assessed [9,12,13]. It has been shown that arbuscular mycorrhizal symbiosis induces changes in secondary compounds, which act as signal molecules in plant–AMF interactions [14]. Investigations of AMF’s effect on aromatic plant biomass [8,15,16], nutrient intake, element composition, biochemical characteristics, accumulation of essential oil and composition revealed significant variability, caused by both the plant species and AMF strain. Genetic variability in AMF populations was shown to affect host-plant fitness [17]. Significant differences in AMF effect on mineral composition and biochemical characteristics were described for garlic and onion [18]. Changes in mineral content of herbs are known to be important factors affecting herb quality [19]. Yield and essential oil composition of *Calamintha nepeta* were shown to be greatly affected by inoculation with different AMF strains [20]. Arbuscular mycorrhizae differentially affect the quality and quantity of essential oils in coriander and dill [21]. 

Due to their widespread use in medicine, the food industry and cosmetics, aromatic plants need to be investigated regarding the effects of AMF on the yield and quality performances of the different species, as well as the economic feasibility of this technology [22,23,24,25].

The quality features of aromatic plants delivered to markets are connected to the biochemical parameters and mineral composition and, in this respect, the effects of beneficial fungi inoculation should be assessed. Moreover, no investigations have been carried out so far on the influence of storage on AMF-inoculated plant characteristics.

The present study was aimed at evaluating the AMF effects on plant growth and development, yield, oil content, biochemical characteristics, elemental composition and the reaction to storage of the aromatic species *A. dracunculus*, *L. angustifolia* and *H. officinalis* grown in open fields.

## 2. Results and Discussion

### 2.1. AMF Effect on Plant Growth, Development and Root Mycorrhizal Colonization, Essential Oil Content, Antioxidant Activity and Phenolics Content

The values relevant to the root mycorrhizal colonization reported in Table 1 are the means of the two determinations performed two months after the transplant and at crop end, as this parameter was stable in the sampled times. AMF occurrence in the roots of the three aromatic species examined was significantly higher under the mycorrhizal-based formulate application, compared to the untreated control, but the inoculation effectiveness was statistically lowest in *Lavandula angustifolia*. Indeed, in the latter crop the beneficial microorganisms did not lead to yield and plant biomass increase, contrary to what was recorded in *Artemisia dracunculus* and *Hyssopus officinalis* (Table 1). In previous research carried out on shallot [26], onion and garlic [18] and tomato [27], AMF inoculation resulted in higher root mycorrhizal colonization percentages than those recorded in the present investigation.

**Figure 1:**
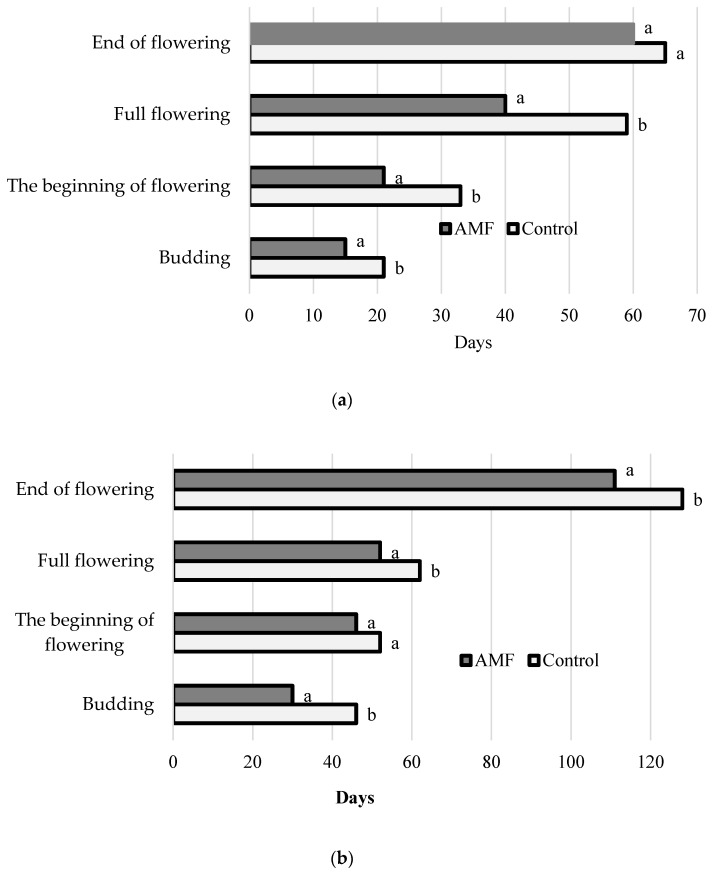

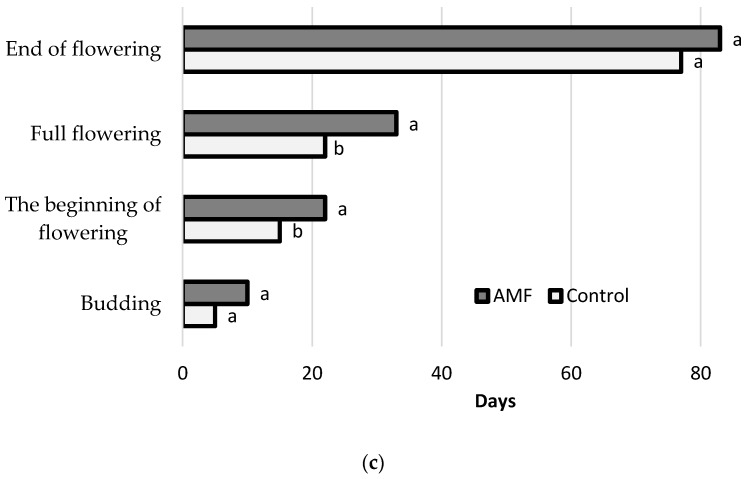
Effect of arbuscular mycorrhizal fungi (AMF) inoculation on aromatic plant phenological development: (**a**) *A. dracunculus*; (**b**) *L. angustifolia*; (**c**) *H. officinalis.* Values followed by different letters are statistically different, according to Duncan’s test, at *p* ≤ 0.05.

The data presented in Figure 1 indicate different effects of AMF preparation on phenological phases of aromatic plant development. Indeed, *A. dracunculus* and *L. angustifolia* inoculated with AMF demonstrated increased rates of development (Figure 1a,b). Conversely, *H. officinalis* showed a slight growth retardation, particularly at the initial stage of development, by 5–8 days.

Despite a slight development slowing, *H. officinalis* demonstrated the highest increase in plant biomass (35.6%), productivity (34.8%) and essential oil yield (33.3%). The latter parameter reached only a 22.4% increase in *A. dracunculus* and 9.3 % in *L. angustifolia*. Though AMF inoculation was reported to elicit plant dry matter accumulation [3], no statistically significant increase in this parameter as well as of total dissolved solids (TDS) were recorded in the present study (Table 1).

According to the literature [16], *Glomus lamellosum* inoculation to *L. angustifolia* resulted in a higher increase in plant biomass (46.7%) and essential oil content (57%) compared to the results from the present investigation, which may be connected with differences in AMF species and soil characteristics. The significant contribution of AMF species for optimizing essential oil accumulation was previously demonstrated in *Artemisia annua* [10]: leaf volatile oil content increased up to 45% and 25% in plants inoculated with *Glomus mosseae* and *Glomus versiforme*, respectively, compared with the control.

The biosynthesis of secondary metabolites in medicinal and aromatic plants depends on genetic, physiological, soil and environmental factors [28] and, in particular, it can be significantly affected by the symbiotic association between arbuscular mycorrhizal fungi (AMF) and roots [29]. A confirmation of the latter phenomenon was reported by Huang et al. [30] upon *Glomus mosseae* inoculation in *Artemisia annua*.

In the present research, among the major essential oil components detected in the aromatic species examined (Table 2; Supplementary Materials), only linalyl acetate in *L. angustifolia* was significantly affected by AMF, which promoted a 34.4% biosynthesis increase. In this respect, the qualitative and quantitative improvement of essential oil production elicits a high commercial interest [9,12].

In previous investigations, compared to the untreated control the AMF inoculation effect on aromatic plant antioxidant activity was positive [31] or not significant [32], the latter report referring to non-stressed conditions. In the present research, lavender, tarragon and hyssop crops were grown under appropriate farming practices, and favorable temperature trends were recorded, which may be supposed as the possible reasons why no significant differences in antioxidant activity and phenolic content were found between AMF-inoculated plants and the untreated control (Table 2). Analysis of the antioxidant status of the aromatic plants revealed that at harvest the antioxidant activity in AMF-treated plants did not significantly differ from the control, being in the range of 58.3 to 58.8 mg GAE g^−1^ for tarragon, 67.9 to 74.7 mg GAE g^−1^ for lavender and 51.6 to 57.0 mg GAE g^−1^ for hyssop. The same situation was recorded for polyphenol content: 18.5–18.6, 19.3–19.8 and 15.0–16.3 mg GAE g^−1^; in contrast with previous findings relevant to the significant effect of AMF on plant antioxidants synthesis [3]. Interestingly, upon storage under stress conditions, AMF inoculation showed significant effects on the quality characteristics of aromatic plants as described in the following section.

### 2.2. Effect of Abiotic Stress

Up to date, in pot experiments carried out in both open fields and greenhouses, the protective effect of AMF against oxidative stress in plants, along with enhanced plant resistance to a range of stresses, including drought, salinity, herbivore, temperature, heavy metals and diseases, were reported [33,34,35]. Indeed, the encouragement of antioxidant status, osmolyte accumulation and selective ion absorption during crop rearing are key tools in maintaining plant tolerance to environmental stresses [36]. However, no attention has been paid to the effect of AMF inoculation on changes in plant antioxidant status during storage. Interestingly, the application of abiotic stress to plants after harvesting, such as a high temperature and/or high humidity during storage of vegetable crops grown in ordinary conditions, usually causes degradation of most natural antioxidants, thus worsening plant quality [37].

**Figure 2:**
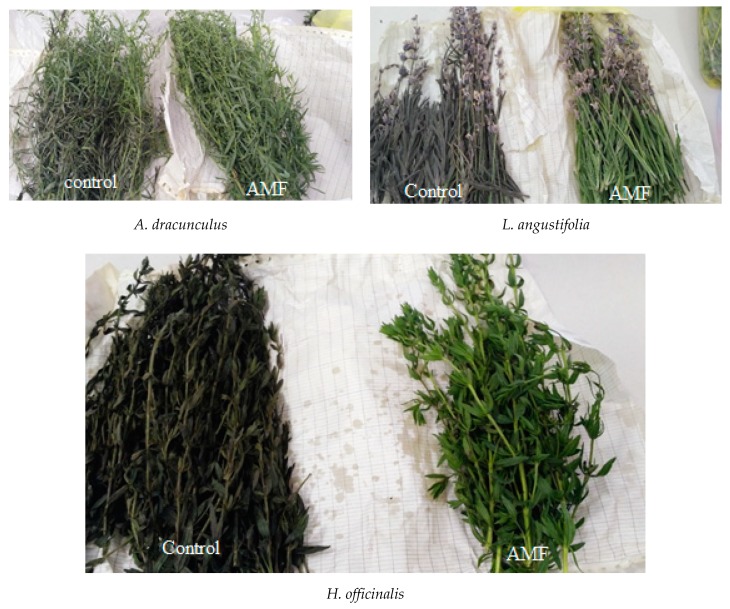
Effect of AMF application on aromatic plant tolerance to abiotic stress.

**Figure 3:**
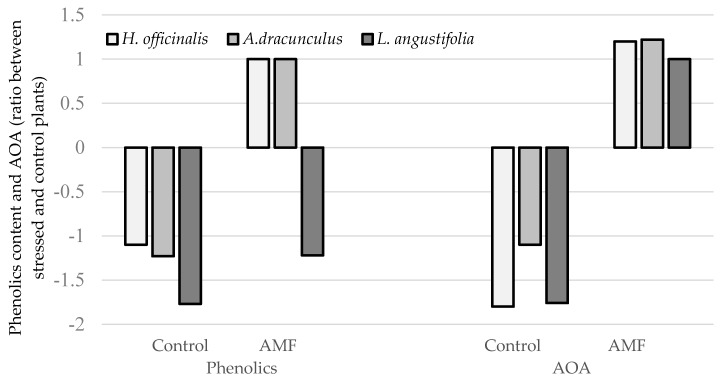
Effect of AMF inoculation on plant phenolic content and AOA of aromatic species (compared to plants not subjected to abiotic stress). AOA, antioxidant activity.

Forced long-term storage of lavender, tarragon and hyssop at high temperature and humidity during the samples transport from Crimea to Moscow, resulted in dramatic changes in plant quality and appearance (Figure 2; Table 3). The results suggest that, despite non-significant differences in antioxidant activity and phenolic content between the AMF-inoculated and control plants at harvest (Table 2), the values of the abovementioned parameters significantly increased during storage in AMF-treated plants compared to the control ones, the latter showing an antioxidant-content drop. The highest differences in appearance and antioxidant status between the AMF-inoculated plants and the control were recorded in hyssop, and the lowest in tarragon (Figure 3). Antioxidant defense plays a major role in protection against plant biotic and abiotic stresses [38,39]. Data presented in Table 3 reveal significantly lower levels of ascorbic acid, chlorophyll, carotenes, phenolics and total antioxidant activity of control plants compared to those inoculated with AMF, which was consistent with the aspect of samples subjected to high temperature and humidity (Figure 2). Notably, the differences between the control and AMF-treated plants in ascorbic acid content reached 1.66 in *A. dracunculus*, 3.64 in *L. angustifolia* and 3.31 in *H. officinalis*. Higher levels of chlorophyll content in the AMF-inoculated plants observed in Figure 1 exceeded those of the control plants by 1.24–2.30 times. Phenolic differences between the AMF-treated and control plants of tarragon and lavender reached 1.42–1.51 times, with similar values recorded in control and AMF-inoculated hyssop.

The comparison between the results obtained on fresh material and those recorded in plants which did not undergo abiotic stress suggests that AMF inoculation provided stability of antioxidant status in most cases, except for polyphenol levels in lavender showing a small content decrease. The most dramatic decrease of AOA due to abiotic stress was revealed in control plants of lavender and hyssop (Figure 3).

The reported protective effect of dry matter against stress tolerance [38] did not occur in the present experiment and even the TDS values did not statistically differ between the AMF-inoculated plants and the control (Table 1). Contrastingly, the content of essential oil, which was shown to have beneficial effects in protecting plants against abiotic and biotic stress [40], increased only in AMF-treated lavender compared to the untreated control (Table 1).

### 2.3. Elemental Composition

**Figure 4:**
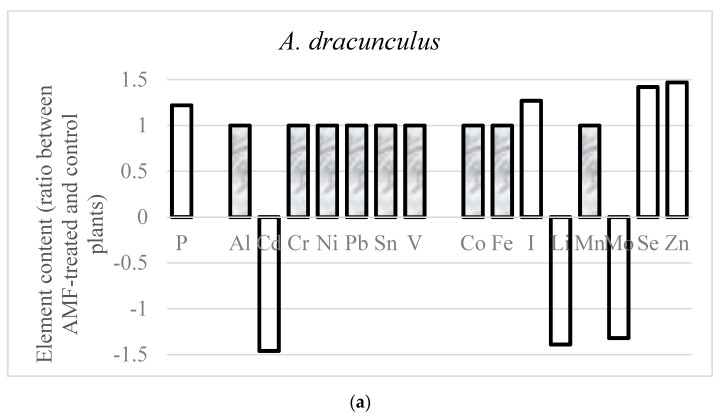

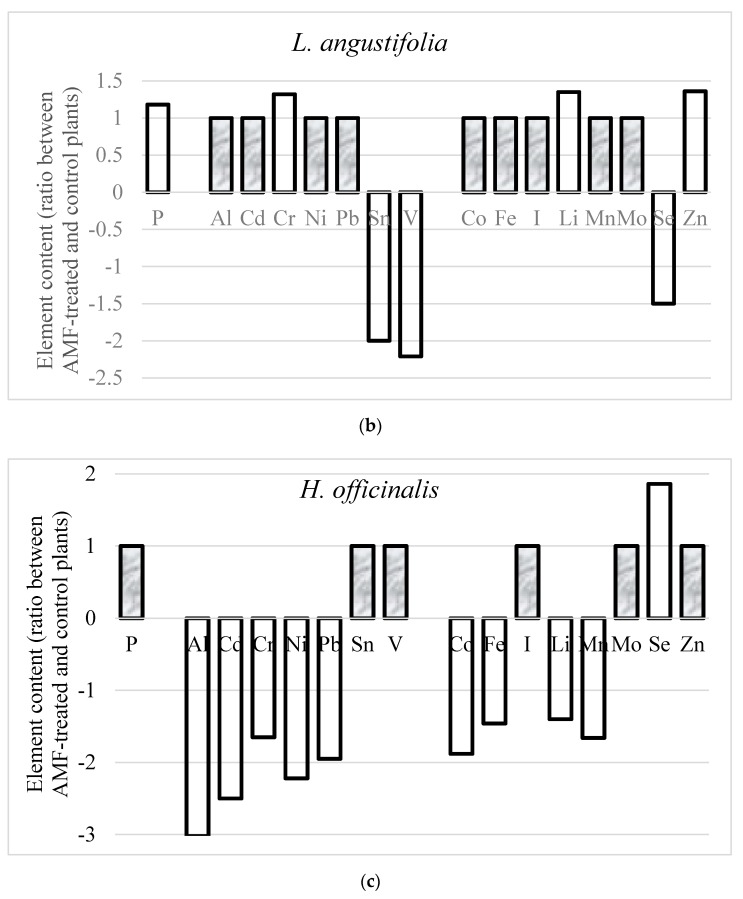
Differences in macro-element, trace-element and heavy metal content between control and AMF-treated plants of *A. dracunculus* (**a**), *L. angustifolia* (**b**) and *H. officinalis* (**c**) (dark columns indicate non-significant differences).

Changes in elemental composition and selective accumulation of specific ions upon AMF inoculation is supposedly a further factor contributing to stress tolerance of the examined aromatic plants. Investigations of AMF effects on macro- and trace-element accumulation in plants revealed an enhanced uptake of nutrients, with particular reference to those characterized by poor mobility in soil (P, Zn, Cu, Fe and Mn) due to the wide AMF hyphae expansion inside the soil [41]. The results of the present research suggest that the effect of AMF inoculation is species-dependent (Figure 4), which is in accordance with previous results relevant to AMF application to garlic and onion [18]. Indeed, AMF inoculation to aromatic plants differently affected the mineral composition of the three crops examined (Table 4, Table 5 and Table 6). Despite the growth stimulating effect of AMF, the concentrations of K, Na, Mg and Ca were not significantly different between the AMF-treated and control plants. Notably, an increase in phosphorous content was recorded in tarragon and lavender, but not in hyssop.

AMF are known to encourage Cu bioavailability [42], but the three plants investigated in the present study did not show any significant changes in this element concentration upon AMF application. Smith and Read [42] also detected an enhanced Zn accumulation in AMF-inoculated plants. In this research, the increase of Zn content was recorded only in tarragon and lavender treated with AMF. A recent investigation of Muszýnska and Labudda [43] showed the ability of Zn to enhance plant tolerance to abiotic stress, which may be partially connected with the increase in stress tolerance of AMF-treated *A. dracunculus* compared to *L. angustifolia* and *H. officinalis* in the present investigation. Interestingly, the concentration of two other natural antioxidants, such as iodine and selenium, significantly increased in AMF-inoculated *A. dracunculus* plants, showing the highest tolerance to abiotic stress as shown by biochemical analysis (Table 5). The reduced concentration of Se in AMF-inoculated lavender compared to control plants, contrary *to A. dracunculus* and *H. officinalis*, suggests that AMF application may species-dependently enhance the concentration of this essential element to human beings. Previously, the beneficial effect of AMF inoculation on Se accumulation in garlic, onion and shallot bulbs was reported [18,26]. Lavender showed a remarkable increase in Cr, Li and Zn content. 

Among the heavy metals (Table 6), AMF only reduced the Cd content in tarragon, Sn and V in lavender and Al, Cd, Cr, Ni and Pb in hyssop plants (Figure 3a–c).

As for the essential elements, Co, Fe, Li and Mn were reduced by AMF treatment in hyssop plants and, similarly, a decrease in Mn concentration was previously recorded in grapevine leaves [44].

AMF-inoculated tarragon plants showed Li and Mo content lowering, whereas no variation was detected for the other elements analyzed.

## 3. Material and Methods

### 3.1. Growth Conditions and Experimental Protocol

The research was conducted at the experimental open field of Nikita Botanic Gardens, situated at the shore of the Black Sea (44°31′ N, 34°15′ E, 200 m above sea level), characterized by a Mediterranean-type dry subtropical climate, with a mean year temperature of 12–15 °C and average daily temperature above 5 °C since the first or second decade of March to the third decade of November (Table 7). The experiment was carried out on an agro-brown, slightly carbonate, light-clay soil with 2.7–3.0% humus, 5.4% carbonates and a pH of 7.8–7.9.

The effects of arbuscular mycorrhizal fungi (AMF)-based formulate application, in comparison with an untreated control, were assessed on plant growth and development; yield; essential oil content and its major components; quality; antioxidant compounds and activity; and elemental composition of the three aromatic species *A. dracunculus* (cultivar Izumrud), *L. angustifolia* (cultivar Record) and *H. officinalis* (cultivar Nikitsky bely). A randomized complete block design was used with three replicates, with the experimental unit surface area of 10 m^2^. A further comparison was performed between plants subjected to abiotic stress (40 °C and 95% relative humidity) during storage and control plants stored in ambient conditions.

The transplant of the three species *A. dracunculus* cultivar Izumrud, *L. angustifolia* cultivar Record and *H. officinalis* cultivar Nikitsky bely was performed on 10 April with the plants spaced 50 cm along the rows that were 50 cm apart for all species examined. The crops were organically managed, in compliance with the EU Regulation 834/2007 and subsequent updates: 50 kg·ha^−1^ N, 17 P_2_O_5_ and 72 K_2_O through organic manure supplied at planting to each species; irrigation was activated when the soil available water dropped to 70%; manual weeding was practiced during crop growing.

The AMF-based formulate Rhizotech MB (Msbiotech S.p.A., Larino, Campobasso, Italy) was applied at 2 g·m^−2^ soil, and it is a plant-growth-stimulating preparation that predominantly contains the endomycorrhizal fungus *Rhizophagus intraradices*, along with low concentrations of *Trichoderma harzianum* and *Bacillus subtilis*. Three AMF inoculations were carried: at planting, on 7 May and on 27 May before the onset of high summer temperatures.

Root mycorrhizal colonization (as a percentage) was assessed twice, two months after planting and at the crop cycle end, according to the Giovannetti and Mosse method [45].

The harvest of the three aromatic species was performed on 4 October, at the end of flowering/beginning of fruiting phase coinciding with the decrease of plant growth rate. At this stage, biometric and yield parameters were assessed according to the methodology carried out at the Department of Aromatic and Medicinal Plants of Nikita Botanic Gardens [46].

### 3.2. Sample Preparation

Plant samples were randomly taken from each plot at harvest and biochemical and elemental analyses were performed on control and AMF-treated plants. Two groups of samples were used: 1) fresh and 2) dry. 1) A fraction of fresh samples in hermetically closed plastic bags was transported by plane (total transport time 8 h) from Crimea to Moscow to the laboratories of the Federal Scientific Center of Vegetable Production, where ascorbic acid, phenolics, photosynthetic pigments and total AOA were assessed. Transport conditions provided significant abiotic stress to harvested plants: temperature in polyethylene bags was about 40 °C and relative humidity 95%. 2) The remaining control samples were dried at room temperature in the shade up to a constant weight, and next homogenized and subjected to quality determinations (antioxidants, total dissolved solids and elemental analysis). All the results were expressed per dry weight.

### 3.3. Dry Residue 

The dry residue was assessed by drying the samples in an oven at 70 °C until a constant weight.

### 3.4. Ascorbic Acid

The ascorbic acid content was determined by visual titration of plant extracts in 6% trichloracetic acid with Tillmans reagent [47]. Three grams of fresh leaves were homogenized in a porcelain mortar with 5 mL of 6% trichloracetic acid and quantitatively transferred to a measuring cylinder. The volume was brought to 60 mL using trichloracetic acid, and the mixture was filtered through filter paper 15 min later. The concentration of ascorbic acid was determined from the amount of Tillmans reagent that went into titration of the sample. 

### 3.5. Polyphenols 

Polyphenols were determined in ethanol extract using the Folin–Ciocalteu colorimetric method as previously described [48]. One gram of dry leaf powder of the aromatic plants was extracted with 20 mL of 70% ethanol at 80 °C over 1 h. The mixture was cooled and quantitatively transferred to a volumetric flask, and the volume was adjusted to 25 mL. The mixture was filtered through filter paper, and 1 mL of the resulting solution was transferred to a 25 mL volumetric flask to which 2.5 mL of saturated Na_2_CO_3_ solution and 0.25 mL of diluted (1:1) Folin–Ciocalteu reagent were added. The volume was brought to 25 mL with distilled water. One hour later the solutions were analyzed through a spectrophotometer (Unico 2804 UV, USA), and the concentration of polyphenols was calculated according to the absorption of the reaction mixture at 730 nm. As an external standard, 0.02% gallic acid was used.

### 3.6. Antioxidant Activity (AOA)

The antioxidant activity of the aromatic plants investigated was assessed using a redox titration method [49] via titration of a 0.01 N KMnO_4_ solution with the ethanolic extract of the plants. The reduction of KMnO_4_ to colorless Mn^+2^ in this process reflects the quantity of antioxidants dissolvable in 70% ethanol. The values were expressed in mg gallic acid equivalents (mg GAE g^−1^ dw). In recent years, the method gained great popularity between biochemists due to simplicity and cheapness [50].

### 3.7. Photosynthetic Pigments

Half a gram of fresh leaf sample was homogenized in a porcelain mortar with 10 mL of 96% ethanol. The homogenized sample mixture was quantitatively transferred to a volumetric flask, bringing the volume to 25 mL and the mixture was filtered through filter paper. The resulting solution was analyzed for Chlorophyll-a, Chlorophyll-b and carotene determination through a spectrophotometer (Unico 2804 UV, USA). Calculation of chlorophyll and carotene concentrations was achieved using appropriate equations [51]:

Ch-a = 13.36A_664_ − 5.19A_649_;

Ch-b = 27.43A_649_ − 8.12A_664_;

C c = (1000A_470_ − 2.13 Ch-a − 87.63 Ch-b)/209;

where A = Absorbance, Ch-a = Chlorophyll a, Ch-b = Chlorophyll b and C c = Carotene.

### 3.8. Essential Oil Extraction and Analysis 

In all species investigated in the stage of full flowering, essential oil content in the aerial part of the plants was determined. For this purpose, 50 g of each dry sample were hydro-distilled in a Clevenger-type apparatus for 2 h and then the percentage and yield of essential oils were calculated [52]. The essential oils were dried over anhydrous sodium sulfate, stored in dark glass vials and kept at 4 °C [53]. The composition of the essential oil was investigated in a gas-chromatograph “Chromatec-Kristall 5000.2” (Russia) with a mass-spectrographic detector. Volatile components were separated on a capillary column CR-5 ms (5%-phenylmethyl-polysiloxane, 0.25 mm × 30 m; 0.25 µm film thicknesses). The temperature of injector and transfer line were set to 250 and 300, respectively. The oven was heated to 75 °C, and subsequently 4.0 °C min^−1^ up to 240; the evaporator temperature was −250 °C. The following conditions were adopted: split ratio 1:25, at flow 1.1 mL min^–1^, with helium as carrier gas, and injection volume of 1 mL of essential oil diluted in dichloromethane (1:300 v/v). The components of the essential oils were identified by comparison of their retention indices relative to (C_8_–C_30_) n-alkanes (Sigma-Aldrich, Switzerland) and Supelco analytical standards (USA), and via comparison of their mass-spectra with those of the NIST 14 mass spectra collection (National Institute of Standards and Technologies, USA).

### 3.9. Total Dissolved Solids (TDS)

TDS were determined in plant water extracts (1 g of dry powder in 50 mL of distilled water) using a portable conductometer TDS-3 (HM Digital, Inc., Seoul, Korea). The results were calculated in mg per g of dry weight.

### 3.10. Elemental Composition

Al, As, B, Ca, Cd, Co, Cr, Cu, Fe, Hg, I, K, Li, Mg, Mn, Na, Ni, P, Pb, Se, Si, Sn, Sr, V and Zn contents in aerated-parts powder samples were assessed using ICP–MS on a quadruple mass-spectrometer Nexion 300D (Perkin Elmer Inc., Shelton, CT 06484, USA) equipped with the 7-port FAST valve and ESI SC DX4 autosampler (Elemental Scientific Inc., Omaha, NE 68122, USA) in the Biotic Medicine Center (Moscow). Rhodium 103 Rh was used as an internal standard to eliminate instability during measurements. Quantitation was performed using an external standard (Merck IV, multi-element standard solution), potassium iodide for the iodine calibration and the Perkin–Elmer standard solutions for P, Si and V, and all the standard curves were obtained at 5 different concentrations. For quality control purposes, internal controls and reference materials were tested together with the samples on a daily basis. Microwave digestion of samples was achieved according to standard method [54] with sub-boiled HNO3 (Fluka #02650 Sigma-Al-drich, Co) in the Berghof SW-4 DAP-40 microwave system (Berghof Products + Instruments GmbH 72800 Eningen, Germany), diluted 1:150 with distilled deionized water. Trace levels of Hg and Sn in samples were not taken into account and, accordingly, they were excluded from the Tables. The instrument conditions and acquisition parameters were: plasma power and argon flow, 1500 and 18 L min^−1^, respectively; aux argon flow, 1.6 L min^−1^; nebulizer argon flow, 0.98 L min^−1^; sample introduction system, ESI ST PFA concentric nebulizer and ESI PFA cyclonic spray chamber (Elemental Scientific Inc., Omaha, NE 68122, USA); sampler and slimmer cone material, platinum; injector, ESI Quartz 2.0 mm I.D/; sample flow, 637 μL min^−1^; internal standard flow, 84 μL min ^−1^; dwell time and acquisition mode, 10–100 ms and peak hopping for all analytes; sweeps per reading, 1; reading per replicate, 10; replicate number, 3; DRC mode, 0.55 mL min^−1^ ammonia (294993-Aldrich Sigma-Aldrich, Co., St. Louis, MO 63103 USA) for Ca, K, Na, Fe, Cr and V, optimized individually for RPa and RPq; STD mode, for the rest of analytes at RPa = 0 and RPq = 0.25.

### 3.11. Statistical Analysis

Data were processed by analysis of variance and mean separations were performed through the Duncan multiple range test, with reference to a 0.05 probability level, using SPSS software version 21. Data expressed as percentages were subjected to angular transformation before processing. 

## 4. Conclusions

AMF inoculation of *A. dracunculus, L. angustifolia* and *H. officinalis* grown in an open field at Nikita Botanic Gardens showed beneficial effects on plant growth, development and essential oil content, though high intraspecies variability in elemental composition was recorded. Moreover, the application of AMF to plants during storage under abiotic stress, i.e., high temperature and humidity, has proved a promising method for improving plant quality, even when no beneficial effects were recorded at harvest of crops reared with appropriate farming practices in normal meteorological conditions.

## Figures and Tables

**Table 1:** Effect of AMF on plant growth and root mycorrhizal colonization, yield, essential oil content and TDS of aromatic species.

Parameter	*A. dracunculus*		*L. angustifolia*		*H. officinalis*	
	Control	AMF	Control	AMF	Control	AMF
Root mycorrhizal colonization (%)	23.8 ± 1.9 ^c^	61.5 ± 3.7 ^a^	24.6 ± 2.8 ^c^	52.3 ± 4.1 ^b^	23.3 ± 2.0 ^c^	62.7 ± 4.0 ^a^
Plant height (cm)	52 ± 2 ^a^	54 ± 2 ^a^	65.5 ± 3.5 ^a^	66 ± 2^a^	72.5 ± 2.5 ^b^	67.5 ± 2.5 ^a^
Plant biomass (g)	285 ± 22 ^b^	340 ± 30 ^a^	390 ± 34 ^a^	360 ± 31 ^a^	295 ± 25 ^b^	400 ± 36 ^a^
Yield (kg m^−^²)	0.86 ± 0.05 ^b^	1.02 ± 0.07 ^a^	0.78 ± 0.04 ^a^	0.72 ± 0.04 ^a^	0.89 ± 0.05 ^b^	1.20 ± 0.08 ^a^
Essential oil content (% fw)	0.68 ± 0.03 ^a^	0.70 ± 0.03 ^a^	1.10 ± 0.70 ^b^	1.30 ± 0.80 ^a^	0.4 ± 0.01 ^a^	0.4 ± 0.01 ^a^
Essential oil yield (g m^−^²)	5.8 ± 0.2 ^b^	7.1 ± 0.3 ^a^	8.6 ± 0.4 ^a^	9.4 ± 0.5 ^a^	3.6 ± 0.1 ^b^	4.8 ± 0.2 ^a^
Dry matter (%)	28.4 ± 1.0 ^a^	29.3 ± 0.3 ^a^	24.6 ± 0.8 ^a^	26.5 ± 1.0 ^a^	26.4 ± 0.7 ^a^	26.8 ± 0.8 ^a^
TDS (mg g^−1^ dw)	58.1 ± 1.4 ^a^	59.8 ± 1.2 ^a^	43.4 ± 1.0 ^a^	45.3 ± 1.0 ^a^	56.7 ± 1.1 ^a^	58.0 ± 1.2 ^a^

fw, fresh weight; dw, dry weight; TDS, total dissolved solids. Along each line and within each species, values followed by different letters are statistically different, according to Duncan’s test, at *p* ≤ 0.05.

**Table 2:** Content of the main essential oil components, phenolics and antioxidant activity in aromatic plants, as affected by AMF inoculation.

Species	Treatment	Essential Oil Component %		AOA mg GAE g^−1^	Phenolics mg GAE g^−1^
*A. dracunculus*	Control	Methyl charvicol	79.3	58.3	18.5
	AMF		81.1	58.8	18.6
			ns	ns	ns
*L. angustifolia*	Control	Linalool	34.6 ^a^	67.9	19.8
		Linalyl aetate	18.8 ^b^		
	AMF	Linalool	36.5 ^a^	74.1	19.3
		Linalyl acetate	25.2 ^a^		
				ns	ns
*H. officinalis*	Control	Isopinocamphone	25.1	57.0a	16.3
		Pinocamphone	10.7		
	AMF	Isopinocamphone	27.2	51.6b	15.0
		Pinocamphone	11.9		
			ns		ns

ns, no statistically significant difference. Within each column and each species, values relevant to the comparison between AMF and the control followed by different letters are statistically different according to Duncan’s test, at *p* ≤ 0.05.

**Table 3:** Effect of AMF inoculation on plant antioxidant compounds and activity of aromatic species subjected to abiotic stress after harvesting.

	*A. dracunculus*		*L. angustifolia*		*H. officinalis*	
	Control	AMF	control	AMF	control	AMF
Ascorbic acid (mg 100 g^−1^)	31.0 ± 0.8 ^a^	51.3 ± 1.4 ^a^	13.4 ± 1.6 ^a^	48.9 ± 1.1 ^b^	14.1 ± 1.0 ^a^	46.8 ± 1.2 ^b^
Chlorophyll a (mg g^−1^)	1.23 ± 0.1 ^b^	3.78 ± 0.2 ^a^	0.83 ± 0.1 ^b^	1.16 ± 0.1 ^a^	1.46 ± 0.1 ^b^	1.77 ± 0.1 ^a^
Chlorophyll b (mg g^−1^)	0.74 ± 0.04 ^a^	0.81 ± 0.05 ^a^	0.55 ± 0.02 ^b^	0.64 ± 0.02 ^a^	0.80 ± 0.05 ^b^	1.04 ± 0.08 ^a^
Carotenes (mg g^−1^)	0.21 ± 0.01 ^b^	0.28 ± 0.01 ^a^	0.14 ± 0.01 ^b^	0.23 ± 0.01 ^a^	0.30 ± 0.01 ^b^	0.33 ± 0.01 ^a^
Phenolics (mg GAE g^−1^ dw)	12.8 ± 0.5 ^b^	19.3 ± 0.8 ^a^	11.2 ± 0.5 ^b^	15.9 ± 0.7 ^a^	15.0 ± 0.7 ^a^	15.0 ± 0.7 ^a^
AOA (mg g^−1^)	53.0 ± 2.0 ^b^	71.8 ± 2.4 ^a^	38.6 ± 1.3 ^b^	73.3 ± 2.5 ^a^	31.5 ± 1.0 ^b^	61.2 ± 2.1 ^a^

Along each line and within each species, values followed by different letters are statistically different according to Duncan’s test, at *p* ≤ 0.05.

**Table 4:** Macro-element content in aromatic plants inoculated with AMF (g·kg^−1^ dw).

Species	Treatment	Ca	K	Mg	Na	P
*A. dracunculus*	Control	21.7 ^a^	29.1 ^a^	4.13 ^a^	1.11 ^a^	4.47 ^a^
	AMF	20.2 ^a^	33.1 ^a^	3.11 ^a^	1.19 ^a^	5.43 ^b^
*L. angustifolia*	Control	12.9 ^a^	31.8 ^a^	5.45 ^a^	0.82 ^a^	3.62 ^a^
	AMF	15.6 ^a^	29.7 ^a^	5.55 ^a^	1.03 ^a^	4.26 ^b^
*H. officinalis*	Control	27.9 ^a^	20.2 ^a^	4.55 ^a^	0.75 ^a^	3.71 ^a^
	AMF	27.9 ^a^	19.8 ^a^	4.19 ^a^	0.78 ^a^	3.74 ^a^

Within each column and species, values followed by different letters are statistically different according to Duncan’s test, at *p* ≤ 0.05.

**Table 5:** Trace elements content in aromatic plants inoculated with AMF (mg·kg^−1^ dw).

Element	*A. dracunculus*		*L. angustifolia*		*H. officinalis*	
	Control	AMF	Control	AMF	Control	AMF
B	39.6 ^a^	43.5 ^a^	14.5 ^a^	15.3 ^a^	16.5 ^a^	16.6 ^a^
Co	0.07 ^a^	0.07 ^a^	0.26 ^a^	0.22 ^a^	0.15 ^a^	0.08 ^b^
Cu	13.2 ^a^	13.4 ^a^	9.2 ^a^	10.2 ^a^	12.0 ^a^	9.8 ^a^
Fe	79.2 ^a^	77.3 ^a^	182.0 ^a^	215.0 ^a^	155.0 ^a^	106.0 ^b^
I	1.13 ^a^	1.44 ^b^	1.04 ^a^	1.23 ^a^	0.9 ^a^	0.92 ^a^
Li	0.99 ^a^	0.71 ^b^	0.31 ^a^	0.42 ^b^	0.21 ^a^	0.15 ^b^
Mn	69.2 ^a^	72.6 ^a^	65.3 ^a^	52.4 ^b^	45.3 ^a^	27.3 ^b^
Mo	0.90 ^a^	0.68 ^b^	1.24 ^a^	1.36 ^a^	1.29 ^a^	1.16 ^a^
Se	0.12 ^a^	0.17 ^b^	0.15 ^a^	0.10 ^b^	0.07 ^a^	0.13 ^b^
Si	3.36 ^a^	3.67 ^a^	3.22 ^a^	3.71 ^a^	3.36 ^a^	3.03 ^a^
Zn	21.6 ^a^	31.7 ^b^	16.0 ^a^	21.7 ^b^	20.4 ^a^	19.7 ^a^

Along each line and within each species, values followed by different letters are statistically different according to Duncan’s test, at *p* ≤ 0.05.

**Table 6:** Heavy metals content in aromatic plants inoculated with AMF (mg·kg^-1^ d.w.).

Element	*A. dracunculus*		*L. angustifolia*		*H. officinalis*	
	Control	AMF	Control	AMF	Control	AMF
Al	33.5 ^a^	36.7 ^a^	107.0 ^a^	132.0 ^a^	89.6 ^a^	29.7 ^b^
As	0.13 ^a^	0.15 ^a^	0.26 ^a^	0.26 ^a^	0.17 ^a^	0.17 ^a^
Cd	0.35 ^a^	0.24 ^b^	0.05 ^a^	0.04 ^a^	0.15 ^a^	0.06 ^b^
Cr	0.29 ^a^	0.24 ^a^	0.59 ^a^	0.78 ^b^	0.56 ^a^	0.34 ^b^
Ni	2.70 ^a^	2.31 ^a^	2.50 ^a^	3.00 ^a^	3.13 ^a^	1.41 ^b^
Pb	0.16 ^a^	0.15 ^a^	0.54 ^a^	0.47 ^a^	0.37 ^a^	0.19 ^b^
Sn	0.03 ^a^	0.04 ^a^	0.04 ^a^	0.02 ^b^	0.04 ^a^	0.03 ^a^
Sr	65.9 ^a^	80.1 ^a^	137.0 ^a^	163.0 ^a^	72.1 ^a^	76.0 ^a^
V	0.44 ^a^	0.43 ^a^	0.31 ^a^	0.14 ^b^	0.15 ^a^	0.16 ^a^

Along each line and within each species, values followed by different letters are statistically different according to Duncan’s test, at *p* ≤ 0.05.

**Table 7:** Values of meteorological parameters relevant to the growing period.

	March	April	May	June	July	August	September
Average daily temperature (°С)	6.9	11.2	17.2	24.8	23.2	24.9	19.9
Minimum temperature (°С)	−1.4	3	7.7	15.9	15	16.6	9.2
Maximum temperature (°С)	17.3	22.2	28	34.7	32.7	36.8	30.6
Sunshine duration (hours)	220	237	258	312	316	317	250
Rainfall (mm)	24.3	43.7	0.9	72.5	21.3	22.3	15.2
Air humidity (%)	64	63	69	58	59	54	58

## Supplementary Materials

The following are available online at https://www.mdpi.com/2223-7747/9/3/375/s1, Table S1: Essential oil composition of *Lavandula angustifolia* Mill. cultivar Record inoculated or non-inoculated with AMF. Table S2: Essential oil composition of *Artemisia dracunculus* L. cultivar Izumrud inoculated or non-inoculated with AMF. Table S3: Essential oil composition of *Hyssopus officinalis* cultivar Nikitsky bely inoculated or non-inoculated with AMF.
