# Supplementary material for: Yield, Essential Oil and Quality Performances of Artemisia dracunculus, Hyssopus officinalis and Lavandula angustifolia as Affected by Arbuscular Mycorrhizal Fungi under Organic Management
Source: Plants (Basel). 2020 Mar 18;9(3):375. doi: 10.3390/plants9030375 (PMC7154847; doi:10.3390/plants9030375)
Supplement: Supplementary file 1 [file plants-09-00375-s001.pdf]

# Supplements

**Table 1.** Essential oil composition of *Lavandula angustifolia* Mill. cultivar Record inoculated and non-inoculated with AMF.

| Compound | Time (min) | Control (%) | AMF (%) |
|---|---|---|---|
| α-Thujene | 6.54 | 0.04 | - |
| α-Pinene | 6.77 | 0.08 | - |
| Camphene | 7.19 | 0.09 | - |
| 1-Octen-3-ol | 7.60 | 0.41 | 0.35 |
| Sabinene | 7.67 | 0.13 | |
| β-Myrcene | 7.95 | 0.85 | 0.45 |
| Hexyl acetate | 8.38 | - | 0.03 |
| α-Phellandrene | 8.53 | 0.11 | - |
| 3-Carene | 8.68 | 0.76 | 0.29 |
| m-Cymene | 8.85 | 0.05 | - |
| p-Cymene | 9.00 | 0.16 | 0.12 |
| trans-β-Ocimene | 9.19 | - | 3.32 |
| Limonene | 9.20 | 3.10 | - |
| Eucalyptol | 9.27 | 8.41 | 3.62 |
| (Z)-Ocimene | 9.53 | 0.65 | 0.69 |
| γ-Terpinene | 10.01 | 0.11 | 0.09 |
| cis-Sabinene hydrate | 10.32 | 0.29 | 0.21 |
| α- Terpinolen | 10.90 | 0.12 | 0.09 |
| Linalool | 11.16 | 34.56 | 36.46 |
| 1-Octen-1-ol, acetate | 11.24 | 0.49 | 0.51 |
| cis-p-Menth-2-en-1-ol | 12.02 | 0.15 | 0.11 |
| (-)-Camphor | 12.76 | 0.23 | 0.21 |
| Lavandulol | 13.19 | 0.42 | 0.40 |
| δ-Terpineol | 13.44 | 0.08 | 0.06 |
| endo-Borneol | 13.58 | 2.62 | 1.80 |
| p-Cymen-8-ol | 13.72 | 0.03 | - |
| Terpinen-4-ol | 13.85 | 6.72 | 8.69 |
| Cryptone | 13.98 | 0.96 | 0.72 |
| α-Terpineol | 14.26 | 3.48 | 3.46 |
| trans-Piperitol | 14.74 | 0.13 | 0.07 |
| Nerol | 15.17 | 0.35 | 0.43 |

| | | | |
|---|---|---|---|
| Cuminal | 15.77 | 0.41 | 0.25 |
| Linalyl acetate | 15.96 | 18.76 | 25.22 |
| (±)-Lavandulyl acetate | 16.99 | 1.59 | 2.26 |
| Thymol | 17.26 | 1.54 | 0.66 |
| Carvacrol | 17.56 | 0.08 | - |
| Neryl acetate | 19.35 | 0.56 | 0.65 |
| Geranyl acetate | 19.95 | 1.44 | 1.39 |
| Santalene | 21.63 | 0.91 | 0.38 |
| β-Caryophyllene | 21.78 | 2.20 | 1.99 |
| α-Bergamotene | 22.04 | 0.25 | 0.09 |
| (E)-β-Famesene | 22.43 | 0.91 | 0.57 |
| Germacrene D | 23.59 | 0.75 | 0.41 |
| γ-Cadinene | 24.51 | 0.76 | 0.41 |
| Caryophyllene oxide | 26.57 | 0.93 | 0.67 |
| Epicubenol | 27.45 | 0.12 | 0.10 |
| .tau.-Cadinol | 28.12 | 2.92 | 2.73 |

**Table 2**. Essential oil composition of *Artemisia dracunculus* L. cultivar Izumrud inoculated or non-inoculated with AMF.

| Compound | Time (min) | Control (%) | AMF (%) |
|---|---|---|---|
| 3-Hexen-1-ol, (Z)- | 4.98 | 0.03 | 0.05 |
| α-Pinene | 6.78 | 0.59 | 0.58 |
| Camphene | 7.17 | - | 0.03 |
| Sabinen | 7.67 | 0.05 | 0.06 |
| β-Pinene | 7.86 | 0.08 | 0.08 |
| β-Myrcene | 7.94 | 0.13 | 0.11 |
| 3-Hexen-1-ol, acetate, (Z)- | 8.20 | 0.09 | 0.04 |
| (E)-Ocimene | 9.19 | 11.90 | 10.78 |
| (Z)-Ocimene | 9.53 | 6.75 | 6.16 |
| α- Terpinolen | 10.89 | 0.07 | - |
| Linalool | 11.08 | 0.04 | 0.06 |
| Neo-allo-ocimene | 12.06 | 0.06 | 0.06 |
| Methyl chavicol | 14.45 | 79.34 | 81.06 |
| Citronellol | 15.16 | 0.04 | - |
| Carvone | 15.82 | - | 0.14 |
| Bornyl acetate | 17.19 | 0.11 | 0.02 |
| Methyleugenol | 20.63 | 0.52 | 0.57 |
| Germacrene D | 23.59 | - | 0.04 |
| Bicyclogermacren | 24.05 | 0.20 | 0.16 |

.

**Table 3.** Essential oil composition of *Hyssopus officinalis* cultivar Nikitsky bely inoculated and non-inoculated with AMF.

| Compound | Time, min | Control, % | AMF, % |
|---|---|---|---|
| α-Thujene | 6.55 | - | 0.03 |
| α-Pinene | 6.79 | 0.06 | 0.09 |
| Sabinene | 7.68 | 0.42 | 0.35 |
| β-Pinene | 7.88 | 2.30 | 2.73 |
| β-Myrcene | 7.96 | 0.28 | tr |
| Limonene | 9.20 | 0.36 | 0.28 |
| Eucalyptol + β-Phellandrene | 9.28 | 0.49 | 0.18 |
| (Z)-Ocimene | 9.54 | 0.24 | 0.16 |
| cis-Sabinene hydrate | 10.32 | 0.10 | 0.07 |
| Linalool | 11.10 | 0.57 | 0.82 |
| Nonanal | 11.18 | tr | 0.16 |
| β-Thujone | 11.80 | 0.33 | 0.30 |
| trans-Pinocarveol | 12.66 | 0.14 | 0.29 |
| (E)-Pinocamphone | 13.28 | 52.78 | 50.43 |
| Isopinocamphone | 13.75 | 25.99 | 26.28 |
| Myrtenol | 14.41 | 3.49 | 3.96 |
| - | 15.96 | | 0.15 |
| Methyl myrtenate | 17.49 | 0.49 | 0.49 |
| Carvacrol | 17.55 | | tr |
| - | 18.39 | 0.07 | 0.05 |
| (-)-β-Bourbonene | 20.65 | 0.98 | 1.54 |
| α-Gurjunene | 21.42 | 0.12 | 0.13 |
| β-Caryophyllene | 21.78 | 0.88 | 0.78 |
| Humulene | 22.83 | 0.09 | 0.08 |
| Alloaromadendrene | 23.03 | 0.72 | 0.85 |
| Germacrene D | 23.59 | 1.85 | 1.50 |
| Bicyclogermacren | 24.05 | 2.58 | 2.10 |
| - | 24.61 | | 0.05 |
| Elemol | 25.41 | 2.27 | 3.04 |
| - | 26.08 | | 0.06 |
| (+)-Spathulenol | 26.37 | 1.20 | 1.92 |
| Caryophyllene oxide | 26.57 | 0.45 | 0.51 |

| | | | |
|---|---|---|---|
| - | 27.21 | 0.18 | 0.18 |
| β-Eudesmol | 28.53 | 0.08 | 0.32 |
| - | 28.75 | 0.28 | 0.13 |
| α-Bisabolol | 29.19 | 0.22 | - |
